# A Wideband Multi-Polarized Microstrip Antenna with High Polarization Isolation Based on Dual-Circular Polarization

**DOI:** 10.3390/mi16111209

**Published:** 2025-10-24

**Authors:** Xuenan Wang, Hongcheng Zhou, Xinhui Wang, Xia Lei, Boyang Hao, Mian Zhong, Chao Zhou

**Affiliations:** 1 College of Aviation Electronics and Electrical, Civil Aviation Flight University of China, Deyang 618307, China; wangxuenan@cafuc.edu.cn (X.W.); leixia@cafuc.edu.cn (X.L.); 2 School of Electrical Engineering, Southwest Jiaotong University, Chengdu 611756, China; zhouhc@swjtu.edu.cn; 3 Chengdu Xphased Technology Co., Ltd., Chengdu 610095, China; wxh541@126.com; 4 College of Air Traffic Management, Civil Aviation Flight University of China, Deyang 618307, China; 13032077472@163.com

**Keywords:** wideband, multi-polarization, high polarization isolation, microstrip antenna

## Abstract

To address the limited overlapping bandwidth across polarization modes in conventional multi-polarized antennas, this paper proposes a wideband multi-polarized microstrip antenna with high polarization isolation. Based on the theory of orthogonal dual-circular polarization synthesis, the proposed antenna achieves left-hand circular polarization (LHCP) and right-hand circular polarization (RHCP) under a single-port excitation mode, and can generate arbitrary linear polarization (LP) by simply adjusting the phase when dual-fed. For verification, a prototype operating at the C-band is designed, fabricated, and measured. The measured results agree well with the simulations. For linear polarization, the measured 10 dB bandwidth ranges from 4 GHz to 8 GHz (relative bandwidth of 66.7%), with polarization isolation exceeding 26 dB. For circular polarization, the measured bandwidth (for 10 dB return loss and 3 dB axial ratio) spans 4.1–8 GHz (relative bandwidth of 64.5%), with polarization isolation greater than 15 dB. The linear polarization gain is slightly higher than the circular polarization gain, with a maximum gain of 4.3 dB. The proposed antenna simultaneously features multi-polarization, a wide bandwidth, a low profile (0.03 λ_0_), and high polarization isolation, which can meet the urgent demand for multi-polarized antennas in modern multi-functional integrated wireless systems, such as communication systems, radar, and unmanned aerial vehicles (UAVs).

## 1. Introduction

Traditional single-polarized antennas have struggled to cope with problems such as tight spectrum resources and complex multipath interference, while multi-polarized antennas have become a key approach for overcoming technical bottlenecks, owing to their flexible support for vertical, horizontal, and other linear polarizations (LP), as well as circular polarization (CP) [1]. In multi-polarized antennas, both linear polarization and circular polarization have irreplaceable values: linear polarization has a low design complexity and forms a stable and reliable transmission advantage in fixed-link communication scenarios; circular polarization plays a crucial role in dynamic communication scenarios (including satellite communication, UAVs, and other motion platforms) due to its physical properties such as anti-multipath fading and the suppression of Faraday rotation caused by the ionosphere [2]. It can effectively solve the problem of polarization mismatch caused by changes in the carrier’s attitude. Meanwhile, broadband capability is also essential for multi-polarized antennas, supporting multi-band work for advanced wireless systems.

Polarization isolation is a key performance indicator of multi-polarized antennas, referring to the degree of signal separation between different polarization modes. High polarization isolation effectively suppresses cross-interference between polarizations and enhances the orthogonality of different polarization channels, ensuring communication quality and system anti-interference capability in dense spectrum reuse scenarios. In contrast, insufficient polarization isolation easily causes multi-user signal crosstalk, making accurate resource scheduling difficult even with the adoption of advanced resource allocation optimization models, such as the HGGO_XCovNet proposed in [3].

In recent years, various polarization reconfiguration technologies have been proposed for multi-polarized antennas, including radio frequency (RF) switch reconfiguration, feed network reconfiguration, and mechanical reconfiguration [4]. A multi-polarization reconfigurable antenna was proposed in [5], which employed eight PIN diodes as RF switches between the excitation source and four dipoles, thereby achieving four linear polarizations (0°, +45°, 90°, −45°) within 2.2–3.1 GHz (relative bandwidth of 34%). However, the number of polarization modes is limited. A wideband high-gain multi-linear polarization reconfigurable antenna was presented in [6], supporting five, seven, and nine linear polarization states by using PIN diodes to control different dipole pairs, but it did not support circular polarization. In [7], a polarization reconfigurable CP planar antenna was implemented, which converted LP waves from a slot antenna into RHCP or LHCP by controlling the states of PIN diodes on the polarizer. However, its overlapping bandwidth is only 1.6% (2.53–2.57 GHz). A broadband quad-polarization reconfigurable omnidirectional antenna was reported in [8], employing 12 PIN diodes in its feeding network to achieve HP, VP, LHCP, and RHCP within 1.65–2.2 GHz (relative bandwidth of 28.6%). However, the large number of PIN diodes leads to complex control and significant challenges in design and optimization. A polarization reconfigurable reflectarray antenna was proposed in [9], which used 3D printing technology to achieve LP, LHCP, and RHCP modes through the mechanical rotation of the reflective panel. However, it suffers from long switching times, complex structure, a large size, and poor integration capability.

Another major implementation method of multi-polarized antennas is dual-port polarization synthesis technology. At present, the most common structure is orthogonal dual-linear polarization, which can be realized through various feeding methods such as aperture coupling, differential feeding, and complementary magnetoelectric coupling [10,11,12,13,14,15]. A linear dual-polarized antenna based on aperture coupling was presented in [10], where electromagnetic waves were coupled from microstrip lines to a square metal structure through U-shaped slots in two directions of the ground plane, thereby exciting linear polarizations in two orthogonal directions, with a relative bandwidth of 4% (3.43–3.57 GHz). A dual-differential-fed, dual-polarized patch antenna was reported in [11], realizing dual-LP through the differential excitation of the antenna’s left–right and upper–lower ends by two ring couplers, with a relative bandwidth of 1.67% (2.38–2.42 GHz). In [12], a dual-LP microstrip patch antenna was implemented in the range of 12.2–13.1 GHz (relative bandwidth of 7.1%) by employing complementary magnetoelectric coupling, where electrical coupling dominated in the x-direction and magnetic coupling in the y-direction. However, this method requires the arrangement of feeding excitations on different layers, resulting in a relatively complex structure. In [15], Zeng et al. proposed a dual-polarized bandwidth-enhanced metasurface antenna, which used two H-shaped slots as a broadband excitation structure and exhibited isolation better than 30 dB in the range of 3.3–5.9 GHz (relative bandwidth of 56.5%).

The orthogonal dual-linear polarization structure is also often used for circular polarization synthesis, but it requires precise amplitude and phase control. For example, the antenna reported in [16] generated HP and VP with single-port excitation, while under dual-port excitation it introduced a ±90° phase difference through a 3 dB coupler and employed a phase shifter to compensate the orthogonally polarized waves, thereby generating LHCP and RHCP. In [17], Kang et al. proposed a quad-polarization reconfigurable microstrip antenna using PIN diodes. In the single-fed mode, the output ports could be switched to produce +45° or −45° LP; in the dual-fed mode, it generated LHCP or RHCP. However, its overlapped bandwidth was only 9.44% (2.32–2.55 GHz). In 2023, Liu et al. proposed a wideband quad-polarization reconfigurable antenna with high isolation based on a switchable feeding network [18], employing only six PIN diodes. For the two LP states, the measured 10 dB bandwidths were 28.4% and 25.3%, while for the two CP states, the measured 3 dB axial ratio bandwidth was 17.4%.

In contrast, orthogonal dual-circular polarization structures remain notably underrepresented in the field of multi-polarized antenna research [19,20,21,22,23], exhibiting far a lower prevalence compared with conventional dual-linear polarization designs. Recent attempts to leverage dual-CP modes for LP synthesis are further constrained by substantial performance limitations and inherent design trade-offs. For instance, the antennas reported in [19,20] only supported discrete polarization angle states (0°, 45°, 90°, 135°) with narrow bandwidths ranging from 5% to 7.87%, while lacking comprehensive experimental validation or demonstrating a limited CP excitation capability. Similarly, studies regarding continuous polarization angle tuning, such as those in [21,22,23], face notable challenges: excessive structural complexity [21], insufficient performance verification [22], or a narrow overlapped bandwidth of 10.53% [23]. Such widespread flaws further highlight the rarity of viable orthogonal dual-CP structures.

The above-mentioned antennas adopting orthogonal dual-port polarization synthesis technology can effectively reduce coupling between ports, but the overlapped bandwidth of linear and circular polarizations remains narrow. Therefore, achieving a wide overlapped bandwidth while enhancing polarization isolation is still a major challenge in the design of multi-polarized antennas.

In this paper, we propose an innovative wideband multi-polarized microstrip antenna with high polarization isolation, designed based on the theory of orthogonal dual-circular polarization synthesis. In contrast to conventional dual-LP synthesis designs, which typically require the strict guarantee of amplitude and phase difference, the proposed antenna can directly achieve CP states with single-port feeding, and only needs to adjust the phases of the two ports when generating LP waves. The measured results match simulations, verifying their multi-polarization capability, wide bandwidth, and high polarization isolation—offering a superior solution for modern multi-functional integrated wireless communication systems.

## 2. Orthogonal Polarization Theory

According to electromagnetic wave theory [24,25], an LP plane electromagnetic wave can be decomposed into two CP waves of equal amplitude but opposite rotation directions. Conversely, two CP waves with equal amplitude and opposite rotation directions can also be combined into an LP wave.

For two equal-amplitude orthogonal CP waves propagating along the z-axis, if the RHCP wave has an initial phase $\varphi_{0}$ leading the LHCP wave, the expression of the synthesized electromagnetic wave can be written as

(1) $$\begin{array}{c} \vec{E}\left(z,t\right)=\vec{x}E_{m}cos\left(\omega t-kz+\frac{\varphi_{0}}{2}\right)cos\left(\frac{\varphi_{0}}{2}\right)\\ +\vec{y}E_{m}cos\left(\omega t-kz+\frac{\varphi_{0}}{2}\right)sin\left(\frac{\varphi_{0}}{2}\right) \end{array}$$

It can be seen that the synthesized electromagnetic wave is an LP wave, and its vibration direction (the angle θ with the x-axis) is determined by the initial phase difference in the circular polarization. The specific relationship is as follows [19,20,21,22,23]:

(2) $$\theta =\frac{\varphi_{0}}{2}$$

Furthermore, if there is a deviation error δ in the initial phase difference $\varphi_{0}$, the synthesized wave is still a strictly LP wave, with only the rotation direction rotated by δ/2. Therefore, the continuous adjustability of the vibration direction of linear polarization can be achieved by continuously adjusting the phase difference in circular polarization. This method has a strong anti-phase drift capability with no need for additional amplitude calibration and is suitable for polarization tracking.

However, in the classic scenario where orthogonal dual-linear polarized waves are combined into a CP wave, the rotation direction of the synthesized CP is fixed. If it is necessary to adjust the polarization rotation direction, the initial phase difference in the linearly polarized waves must be abruptly changed from 90° to −90°, or conversely. This involves a high parameter sensitivity, requiring the strict guarantee of amplitude and phase difference, thus resulting in very limited regulation of polarization modes.

Therefore, this paper does not adopt the traditional orthogonal dual-linear polarization synthesis theory but instead employs the orthogonal dual-circular polarization synthesizing linear polarization theory. In particular, for the synthetic linearly polarized wave, polarization mismatch is prone to occur when the position and orientation of the transmitting and receiving antennas change due to rapid changes in carrier attitude or moving targets. The backend can track and match the antenna polarization mode by obtaining the tracking error after the polarization mismatch is detected and adjusting the phase difference between LHCP and RHCP in real time to twice the target linear polarization azimuth angle, thereby maintaining a stable communication link and detection performance.

## 3. Antenna Design and Analysis

### 3.1. Antenna Structure

**Figure 1:**
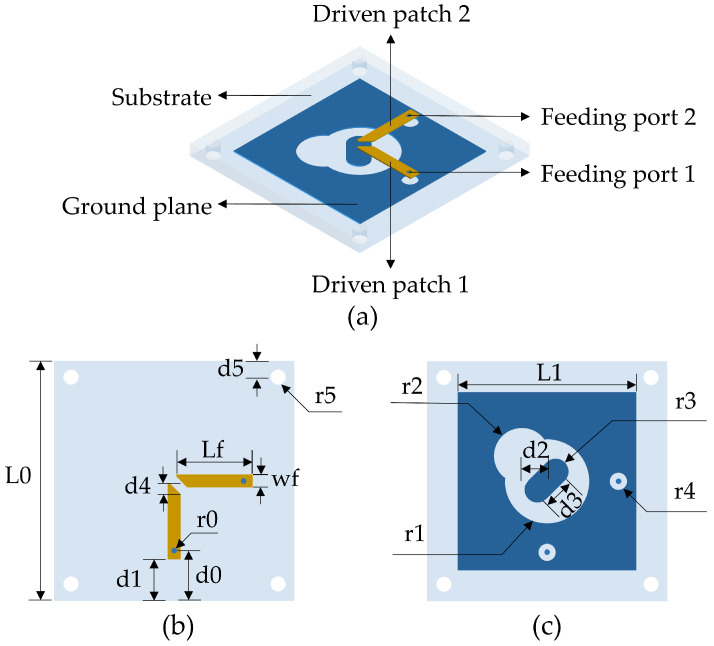
Geometry of the proposed antenna: (**a**) Three-dimensional view. (**b**) Top view. (**c**) Bottom view.

Based on the orthogonal dual-circular polarization synthesis theory, this paper adopts a dual-port coaxial feeding structure and proposes a wideband multi-polarized microstrip antenna with high polarization isolation. Figure 1a shows the structure of the proposed antenna, which consists of driven patch 1 and driven patch 2, a ground plane with slots, and a square dielectric substrate. The proposed antenna is implemented on the substrate of PTFE with a glass reinforced RF circuit material, with a dielectric constant of 2.55 and a thickness of 1.5 mm. To achieve different polarizations in the same frequency band, the antenna employs a symmetrical structure design. The two orthogonal 50-ohm microstrip driven patches are used as feeding ports at the same time to ensure that the antenna naturally meets the orthogonal polarization condition in physical space. The corresponding detailed dimensions are given in Table 1.

### 3.2. Antenna Analysis

**Figure 2:**
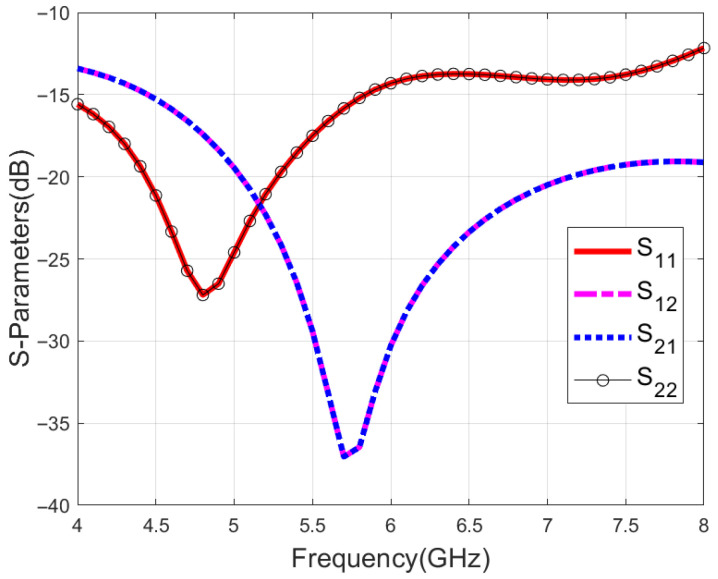
Simulated S-parameters of the proposed antenna.

All the simulations in this paper have been performed by using High-Frequency Structure Simulator (HFSS) 2023. Figure 2 illustrates the reflection coefficient S_11_ of the proposed antenna as well as the isolation between the two ports. Due to the symmetry of the structure, S_11_ is identical to S_22_ and S_21_ is identical to S_12_. The bandwidth for S_11_ of less than −10 dB can reach 66.7% (4–8 GHz). The isolation between the two ports is more than 10 dB, indicating that the two ports are well isolated.

**Figure 3:**
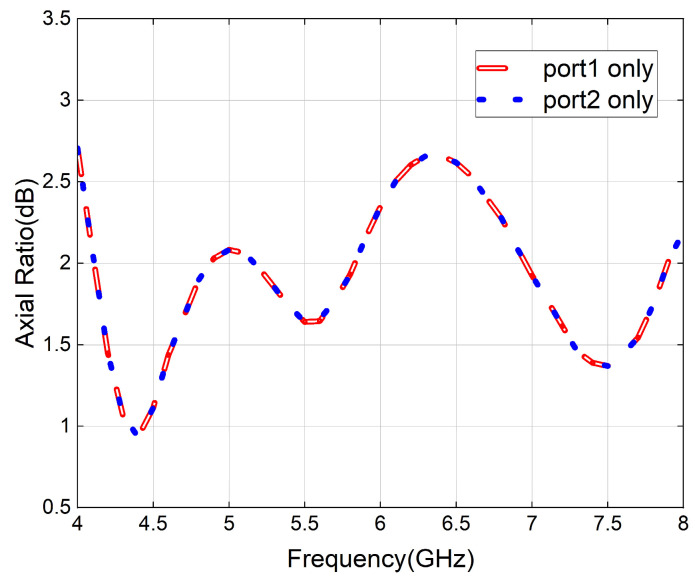
Simulated axial ratio of the proposed antenna.

The axial ratio of the proposed antenna is shown in Figure 3. Regardless of whether port 1 or 2 is utilized to feed the antenna, the value of the axial ratio remains less than 3 dB, and the 3 dB axial ratio bandwidth is 66.7% (4–8 GHz), indicating that the antenna has circular polarization characteristics over a wide frequency band. Given the symmetry of the antenna structure, only the results of circular polarization for the independent feeding of port 1 and linear polarization with a phase difference of 0° will be presented in the following text.

**Figure 4:**
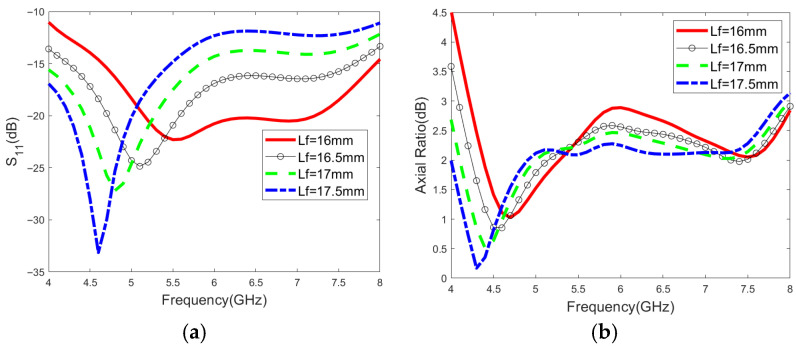

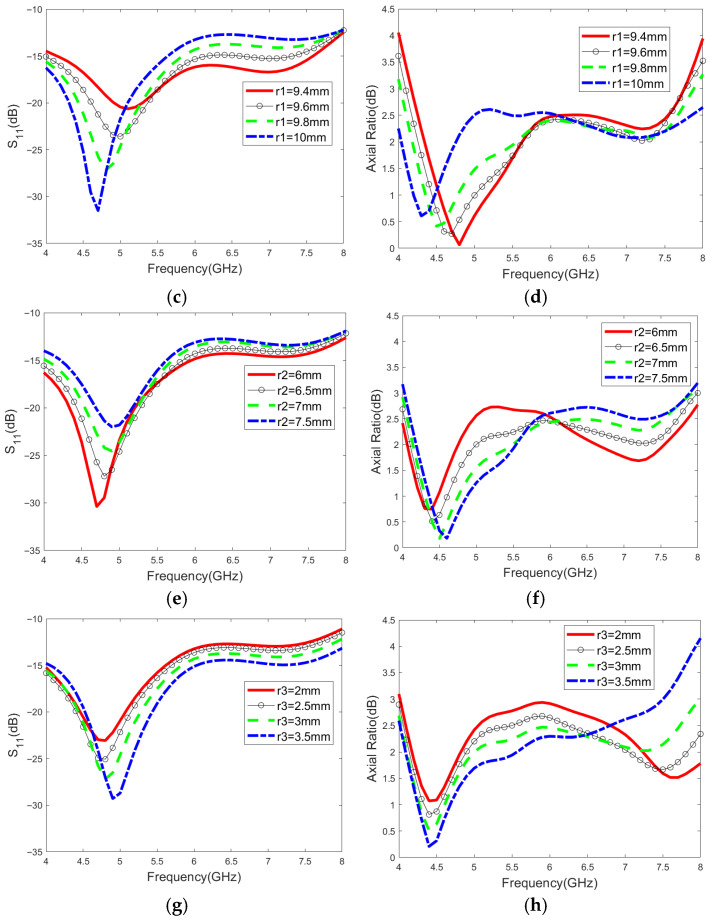
Effects of parameters on the proposed antenna: (**a**) Reflection coefficient with Lf. (**b**) Axial ratio with Lf. (**c**) Reflection coefficient with r1. (**d**) Axial ratio with r1. (**e**) Reflection coefficient with r2. (**f**) Axial ratio with r2. (**g**) Reflection coefficient with r3. (**h**) Axial ratio with r3.

The impact of the significant parameters on the matching performance and circular polarization characteristics is shown in detail in Figure 4. In the case of the single-port excitation mode, the circular polarization characteristics are excited by the interaction between two microstrips and the slotted ground plane, while shortening the length of the microstrip can broaden the impedance bandwidth. Therefore, the length of the microstrip and the sizes of the slots are important parameters for adjusting the matching performance and circular polarization characteristics. As shown in Figure 4a–d, the S_11_ parameter changes significantly with the adjustment of the Lf and r1 values. As illustrated in Figure 4e–h, the variation trend of the axial ratio across the entire frequency band becomes more pronounced with the increase in r2 and r3.

## 4. Experimental Results

**Figure 5:**
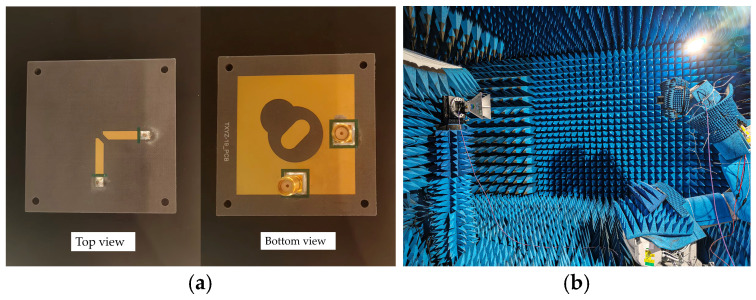
(**a**) Photograph and (**b**) far-field measurement of the antenna prototype.

As shown in Figure 5, the proposed antenna is fabricated with two SMAs and tested in a microwave anechoic chamber.

**Figure 6:**
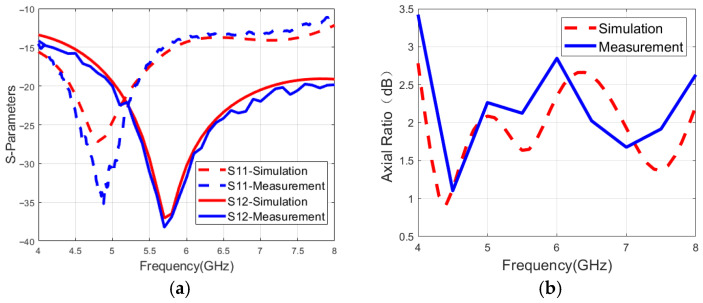
Simulated and measured results: (**a**) S-parameters. (**b**) Axial ratio.

Figure 6a shows the simulated and measured S_11_ and S_12_. The measured results agree well with the simulated data. The measured impedance bandwidth of the proposed antenna simultaneously covers the C-band. The simulated and measured isolation is higher than 10 dB within the 4–8 GHz band. The discrepancy between the simulated and measured S-parameters is mainly caused by the height error during soldering.

Figure 6b plots the simulated and measured axial ratio results when port 1 is fed in an anechoic chamber environment. The measured results have a good agreement with the simulations. The maximum measured axial ratio of the proposed antenna within the 4.1–8.0 GHz band is 3 dB. However, the measured results are slightly different from the simulation results because of the influence of SMA joint welding and the test environment.

**Figure 7:**
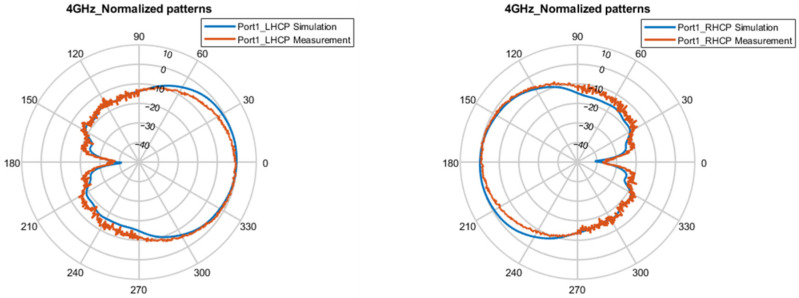

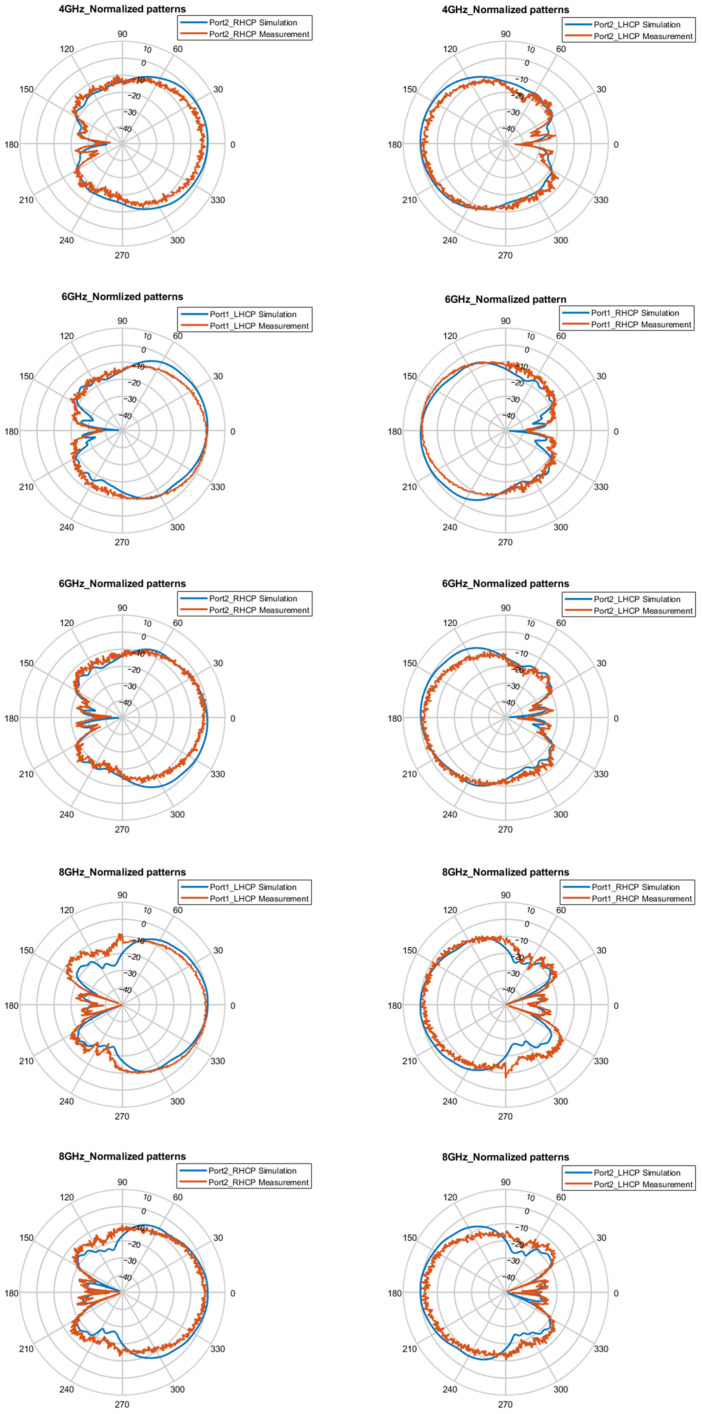
Normalized patterns of E-plane for the CP states at 4, 6, and 8 GHz.

**Figure 8:**
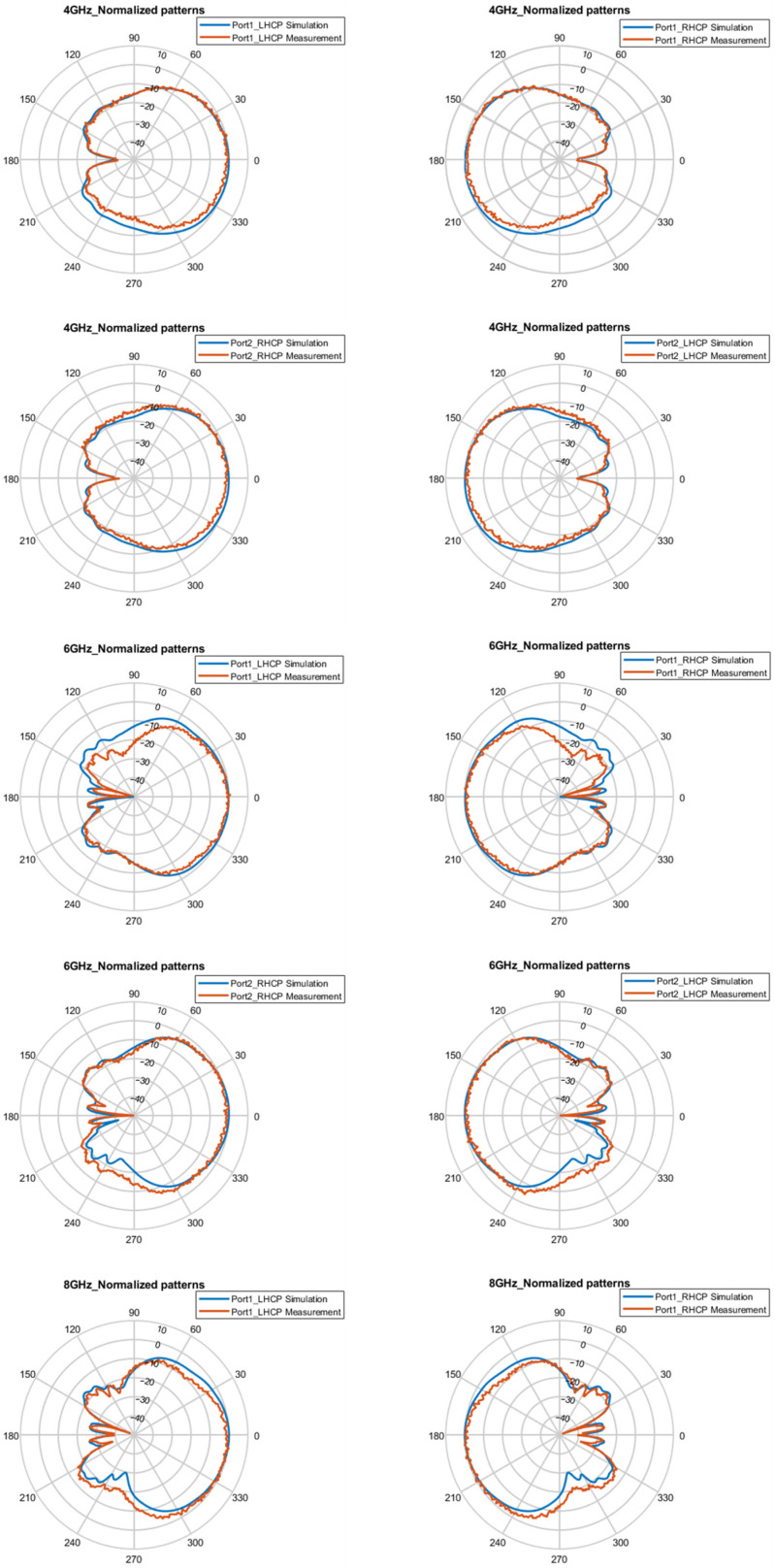

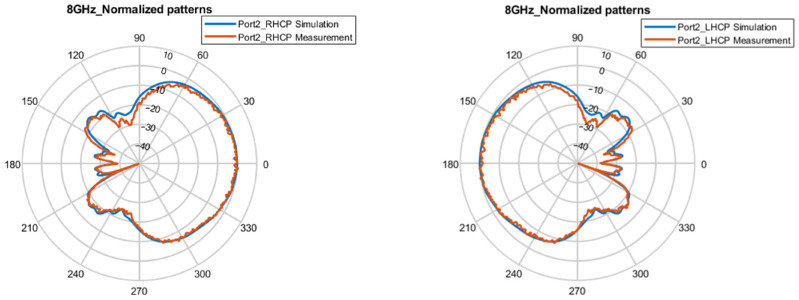
Normalized patterns of H-plane for the CP states at 4, 6, and 8 GHz.

Figure 7 and Figure 8, respectively, present the simulated and measured normalized patterns of the E-plane and H-plane at 4 GHz, 6 GHz, and 8 GHZ under single-port feed conditions. The proposed antenna achieves LHCP in the +z direction and RHCP in the −z direction for port 1 excitation, which is exactly the opposite of the situation when only port 2 is fed. The measured results show that the pattern characteristics are stable and basically consistent with the simulation results, with no obvious distortion in the range of 4–8 GHz.

**Figure 9:**
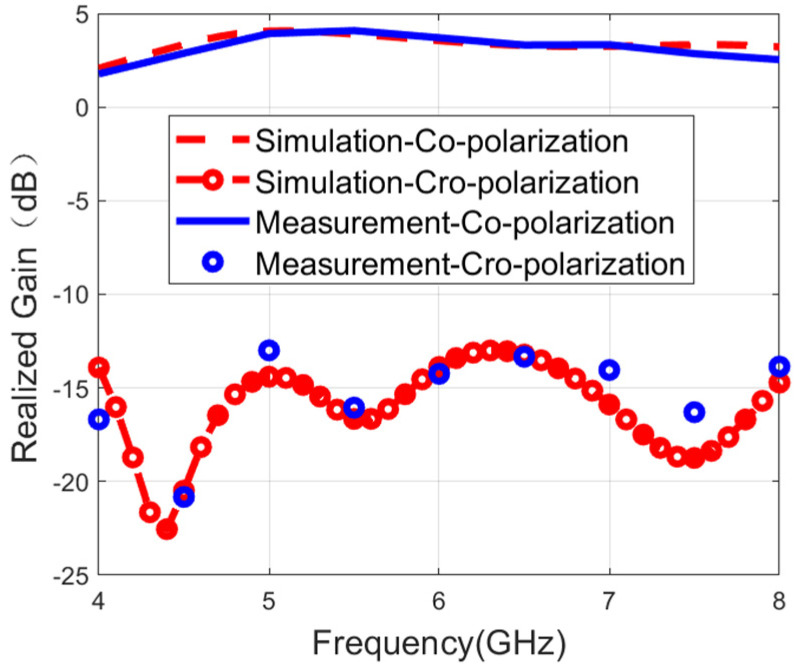
Measured and simulated gain when port 1 is fed.

**Figure 10:**
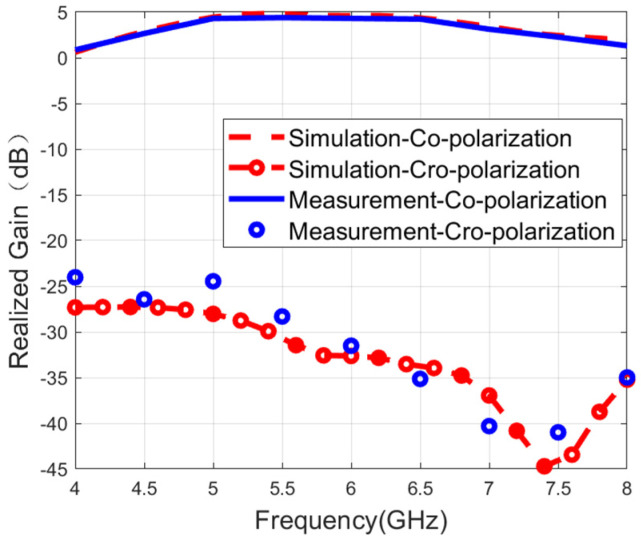
Measured and simulated gain when both ports are fed.

Figure 9 and Figure 10 show the measured and simulated realized gain results. The measured results have a good agreement with the simulations. During the single-port excitation mode, the maximum gain reaches 4.1 dB. The peak gain is 4.3 dB in the case of the dual-fed mode. This phenomenon of relatively low gain is mainly caused by two core characteristics: the wide bandwidth feature and the bidirectional radiation characteristics.

**Figure 11:**
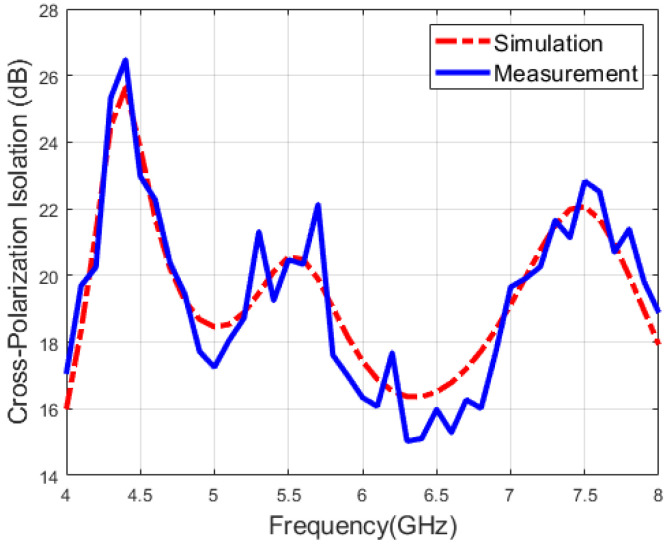
Measured and simulated cross-polarization isolation when port 1 is fed.

**Figure 12:**
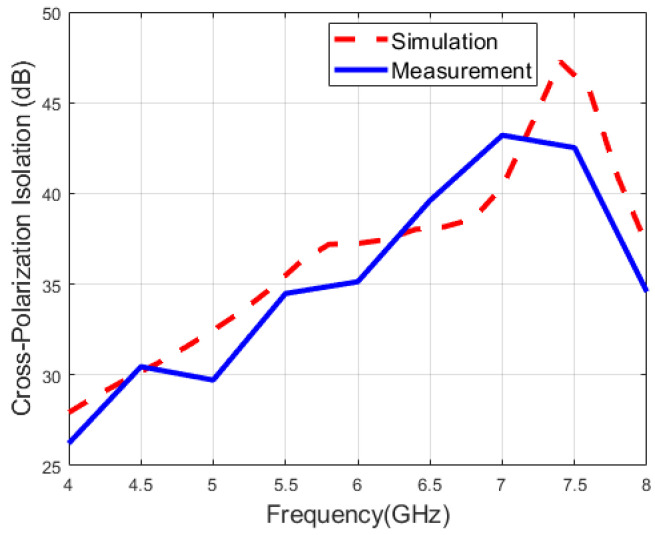
Measured and simulated cross-polarization isolation when both ports are fed.

Figure 11 and Figure 12 illustrate the measured and simulated cross-polarization isolation of the proposed antenna, respectively. During the single-port excitation mode, the cross-polarization isolation is above 15 dB, while the cross-polarization isolation is greater than 26 dB under the dual-fed mode. Namely, the polarization isolation of both polarization modes is relatively high, indicating excellent polarization purity and meeting the application requirements of modern multi-functional integrated wireless systems for multi-polarized antennas.

**Figure 13:**
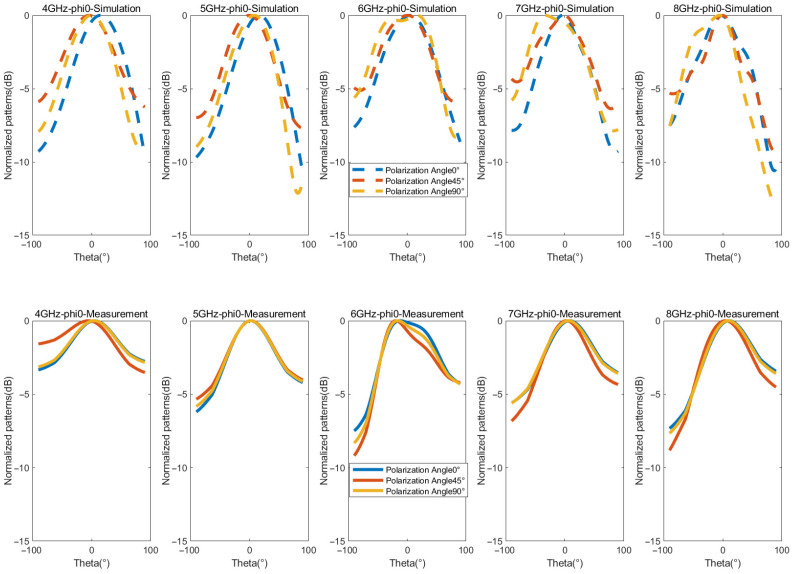
Measured and simulated normalized patterns of E-plane for the LP states.

**Figure 14:**
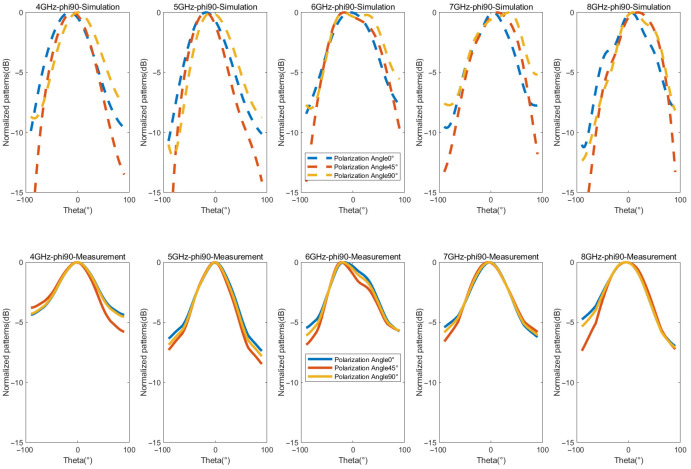
Measured and simulated normalized patterns of H-plane for the LP states.

Figure 13 and Figure 14 present the simulated and measured normalized radiation patterns of the antenna in the E-plane and H-plane at 4, 5, 6, 7, and 8 GHz. According to Formula (2), arbitrary polarization angles can be obtained by adjusting the phase difference between LHCP and RHCP. Here, polarization angles of 0°, 45°, and 90° are taken as examples for illustration. A minor discrepancy between the measured and the simulated results is observed, which can be attributed to the signal attenuation introduced by the controlled tunable phase shifters configured with CP phase differences of 0°, 90°, and 180°. Nevertheless, their overall trends are roughly consistent, indicating that the antenna exhibits stable polarization performance for 0° LP, 45° LP, and 90° LP at the aforementioned frequencies. These measured radiation patterns fully confirm that the proposed antenna can effectively generate arbitrary LP waves by adjusting the phase.

**Figure 15:**
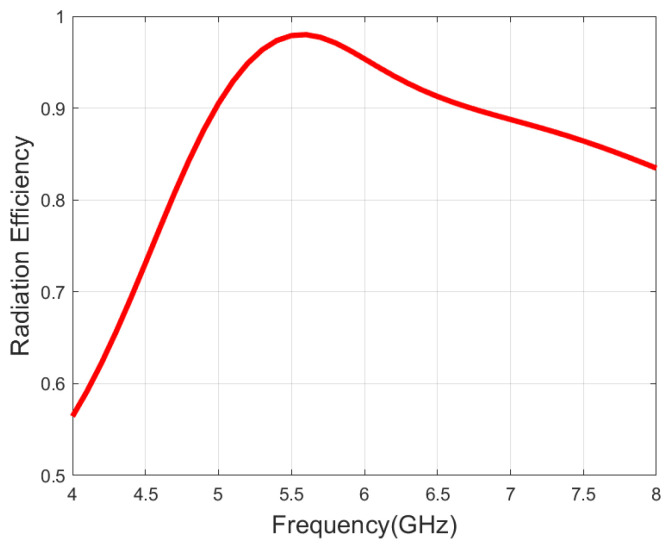
Simulated radiation efficiency.

Figure 15 presents the simulated radiation efficiency of the antenna across the operating band for CP. The efficiency exceeds 90% within the frequency band of 5–6.7 GHz; however, it decreases at the edge frequencies, especially in the low-frequency band. This is mainly attributed to the wideband structure of the antenna, which in turn leads to low gain in the low-frequency band.

Table 2 compares the proposed antenna with reported multi-polarized antennas in terms of polarization modes, the implementation method, the overlapped bandwidth, the polarization isolation, etc. As can be seen, the proposed antenna achieves multi-polarization, a wide bandwidth, and high polarization isolation. Moreover, the proposed antenna exhibits an absolute advantage in terms of bandwidth.

## 5. Conclusions

In this paper, a wideband multi-polarized reconfigurable microstrip antenna with high polarization isolation and an orthogonal polarization-symmetric structure is proposed. The overlapped effective operating frequency band across the three polarization modes reaches 3.9 GHz, corresponding to a relative bandwidth of 64.5%. With advantages such as structural simplicity, a low profile, arbitrary linear polarization, a wide bandwidth, and high polarization isolation, the proposal can effectively reduce equipment redundancy and installation costs.

To further enhance the proposed antenna’s gain and S_11_/AR bandwidth, two core technical paths are proposed: (1) integrating a metasurface with specifically designed apertures above the radiating layer to optimize impedance matching, radiation efficiency, and overall performance [26]; (2) exploring array feeding systems (e.g., sequential phase and orthogonal feeding) via Both-Sided MIC Technology [27]. These designs will be validated through subsequent simulations and experimental measurements for wideband, multi-polarized applications.

Additionally, the integration of active circuits can be considered to be incorporated in future work. Engineering applications such as polarization tracking and polarization matching can be realized by leveraging cross-polarization compensation based on the reconfigurable dual-circular polarization characteristics, thereby significantly improving the environmental adaptability in multi-functional applications including communication systems, radar, and unmanned aerial vehicles (UAVs).

## Figures and Tables

**Table 1:** Detailed dimensions of the proposed antenna.

Parameter	Value (mm)	Parameter	Value (mm)
L0	56	d4	2
L1	41.6	r1	9.8
Lf	17.7	r2	6.5
wf	3	r3	3
d1	9.3	d2	6
d0	11	d3	6
r4	2.05	r0	0.65
r5	1.75	d5	4

**Table 2:** Performance comparison of multi-polarized microstrip antennas.

Ref.	Polarization Modes	Implementation Method	Polarization Isolation/dB	Frequency/GHz	Relative Bandwidth	Overlapped Bandwidth
[8]	VP/HP LHCP/RHCP	polarization reconfiguration (PIN)	20 14	1.65–2.25 1.65–2.2	30.7% 28.6%	28.6%
[9]	LP LHCP/RHCP	polarization reconfiguration (mechanically)	20 15	24–38 26–38	45.2% 37.5%	37.5%
[14]	VP/HP	dual-linear polarization	20	5.15–7.50	37.2%	37.2%
[17]	±45° LHCP/RHCP	dual-linear polarization	18.7 15.6	2.29–2.55 2.32–2.55	10.74% 9.45%	9.45%
[18]	VP/HP LHCP/RHCP	dual-linear polarization	15 14.3	1.66–2.05 1.68–2.00	21.02% 17.39%	17.39%
[21]	LP LHCP/RHCP	dual-circular polarization	20	4.7–5.4 4.8–5.4	13.86% 11.76%	11.76%
[23]	LP LHCP/RHCP	dual-circular polarization	--	2.34–2.66 2.29–2.60	12.8% 12.68%	10.53%
This work	LP LHCP/RHCP	dual-circular polarization	26 15	4–8 4.1–8	66.7% 64.5%	64.5%

## Data Availability

The data presented in this study are available on request from the corresponding author.

## References

[1] Gao S., Sambell A., Zhong S. (2006). Polarization-agile antennas. IEEE Antennas Propag. Mag..

[2] Khan I., Qi X., Shao Y., Zhang K., Song C., Kamal M.M., Wu Q. (2025). Hybrid technique-based circularly polarized MIMO antenna with low mutual coupling for millimeter-wave communications. Opt. Express.

[3] Kamal M.M., Khan I., Al-Khasawneh M.A., Saudagar A.K.J. (2025). Hybrid optimization-based deep learning for energy efficiency resource allocation in MIMO-enabled wireless networks. Sci. Rep..

[4] Sharma S.K., Chieh J.-C.S. (2021). Multifunctional Antennas and Arrays for Wireless Communication Systems.

[5] Wong H., Lin W., Huitema L., Arnaud E. (2017). Multi-polarization reconfigurable antenna for wireless biomedical system. IEEE Trans. Biomed. Circuits Syst..

[6] Chen D., Liu Y., Chen S.-L., Qin P.-Y., Guo Y.J. (2020). A wideband high-gain multilinear polarization reconfigurable antenna. IEEE Trans. Antennas Propag..

[7] Li W., Gao S., Cai Y., Luo Q., Sobhy M., Wei G., Xu J., Li J., Wu C., Cheng Z. (2017). Polarization-reconfigurable circularly polarized planar antenna using switchable polarizer. IEEE Trans. Antennas Propag..

[8] Cui Y., Qi C., Li R.L. (2019). A Low-profile broadband quad-polarization reconfigurable omnidirectional antenna. IEEE Trans. Antennas Propag..

[9] Mei P., Zhang S., Pedersen G.F. (2020). A wideband 3-D printed reflectarray antenna with mechanically reconfigurable polarization. IEEE Antennas Wirel. Propag. Lett..

[10] Ouadefli M., Tribak A., Et-Tolba M., Garcia J.A., Tizyi H. (2022). Linear dual-polarized microstrip patch antenna with u-shaped aperture. Proceedings of the 2022 2nd International Conference on Innovative Research in Applied Science, Engineering and Technology (IRASET).

[11] Nawaz H., Tekin I. (2017). Double-differential-fed, dual-polarized patch antenna with 90 dB interport RF isolation for a 2.4 GHz in-band full-duplex transceiver. IEEE Antennas Wirel. Propag. Lett..

[12] Yu H.W., Jiao Y.C. (2019). Complementary Magneto-Electric Coupling Feeding Methods for Low-Profile High-Isolation Dual-Polarized Microstrip Patch Antenna. Proceedings of the 2019 International Symposium on Antennas and Propagation (ISAP).

[13] Lin F.H., Chen Z.N. (2020). Resonant metasurface antennas with resonant apertures: Characteristic mode analysis and dual-polarized broadband low-profile design. IEEE Trans. Antennas Propag..

[14] Zhang X., Zhou D., Li Y., Wei K., Zhang Z. (2022). A Simple dual-polarized patch antenna array for Wi-Fi 6/6E application. IEEE Trans. Antennas Propag..

[15] Zeng W.F., Chen F.C. (2024). Design of Dual-Polarized Bandwidth-Enhanced Metasurface Antenna Using Characteristic Mode. IEEE Antennas Wirel. Propag. Lett..

[16] Zhao Y., Zhu B., Ding J., Li S. (2024). A Whole W-Band Multi-Polarization Horn Antenna Based on Boifot-Type OMT. Micromachines.

[17] Kang L., Li H., Tang B., Wang X., Zhou J. (2021). Quad-polarization-reconfigurable antenna with a compact and switchable feed. IEEE Antennas Wirel. Propag. Lett..

[18] Liu M., Zhai Z.J., Lin F., Sun H.J. (2023). Wideband quad-polarization-reconfigurable bidirectional antenna with a simple wideband switchable feeding network. IEEE Antennas Wirel. Propag. Lett..

[19] Liu M., Li Z., Zhu C., Cui Z., Ma H. (2022). A Novel Linearly Polarized Antenna with Reconfigurable Polarization Angle. Proceedings of the 2022 International Conference on Microwave and Millimeter Wave Technology (ICMMT).

[20] Lu H.-C., Siao S.-Y., Chuang S.-K., Rao P.-Z., Tung W.-S. (2014). Antenna with switchable linear polarization for 60 GHz. Proceedings of the 2014 IEEE Antennas and Propagation Society International Symposium (APSURSI).

[21] Sano M., Higaki M. (2019). A linearly polarized patch antenna with a continuously reconfigurable polarization plane. IEEE Trans. Antennas Propag..

[22] Shi H., Chen B., Chen M., Sun K. (2022). A polarization reconfigurable antenna with continuously tunable linear polarization angle. Proceedings of the 2022 IEEE MTT-S International Microwave Workshop Series on Advanced Materials and Processes for RF and THz Applications (IMWS-AMP).

[23] Wang S., Yang D., Geyi W., Zhao C., Ding G. (2021). Polarization-reconfigurable antenna using combination of circular polarized modes. IEEE Access.

[24] Balanis C.A. (2016). Antenna Theory: Analysis and Design.

[25] Xie C.F., Rao K.J. (2006). Electromagnetic Fields and Waves.

[26] Anelli F., Loconsole A.M., Losito R., Prudenzano F. (2025). Broadband Circularly Polarized Antenna Array via Metasurface and Partially Emptied Substrate. IEEE Access.

[27] Ushijima Y., Nishiyama E., Aikawa M. (2011). Dual-polarized microstrip array antenna with orthogonal feed circuit. Proceedings of the 2011 IEEE International Symposium on Antennas and Propagation (APSURSI).

